# 633. Incidence and Outcomes of Pediatric Allogeneic Hematopoietic Cell Transplant (allo-HCT) Recipients Under Surveillance and Not Under Surveillance for Human Adenovirus (HAdV) in the Post-HCT Period

**DOI:** 10.1093/ofid/ofae631.198

**Published:** 2025-01-29

**Authors:** Jesse A Blumenstock, Craig Boge, Sydney Shuster, Yun Li, Alix E Seif, Michael D Green, Marian G Michaels, Jessie L Alexander, Monica I Ardura, Jeffrey Auletta, Tamara P Miller, Diego R Hijano, William J Muller, Jennifer E Schuster, Abby M Green, Daniel Dulek, Adriana E Kajon, Michael Grimley, Lara A Danziger-Isakov, Brian T Fisher

**Affiliations:** Children's Hospital of Philadelphia, Philadelphia, PA; Children's Hospital of Philadelphia, Philadelphia, PA; Children's Hospital of Philadelphia, Philadelphia, PA; University of Pennsylvania, Philadelphia, Pennsylvania; Children's Hospital of Philadelphia, Philadelphia, PA; University of Pittsburgh School of Medicine, Pittsburgh, PA; UPMC Children's Hospital of Pittsburgh, Pittsburgh, Pennsylvania; Stanford University School of Medicine, Palo Alto, California; Nationwide Children's Hospital, Columbus, OH; The Ohio State University, Columbus, OH; Emory University/Children's Healthcare of Atlanta, Atlanta, Georgia; St. Jude Children's Research Hospital, Memphis, TN; Ann and Robert H. Lurie Children’s Hospital of Chicago and Northwestern University Feinberg School of Medicine, Chicago, Illinois; Children’s Mercy Kansas City, Kansas City, Missouri; Washington University School of Medicine, St. Louis, Missouri; Vanderbilt University Medical Center, Nashville, Tennessee; Lovelace Biomedical Research Institute, Albuquerque, New Mexico; University of Cincinnati College of Medicine, Cincinnati, Ohio; Cincinnati Children's Hospital, Cincinnati, Ohio; Children’s Hospital of Philadelphia, Philadelphia, Pennsylvania

## Abstract

**Background:**

Human Adenovirus (HAdV) can cause life-threatening illness in pediatric allo-HCT recipients, but studies to define incidence and outcomes of HAdV in allo-HCT patients are limited. Use of PCR-based HAdV surveillance varies by HCT center, in part because there are no approved HAdV-directed therapies. This study describes the epidemiology of HAdV infection and disease in pediatric allo-HCT patients who did and did not undergo HAdV surveillance.

**Figure:**
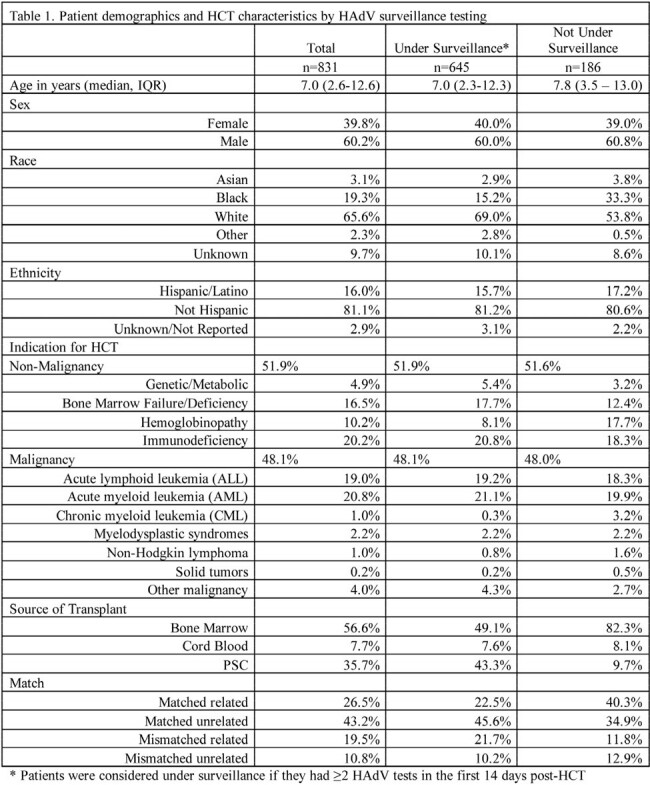

**Methods:**

An allo-HCT cohort was assembled from July 2018 to June 2021 at ten US pediatric HCT centers. Patients were followed for 180 days from HCT to document presence of HAdV infection and disease and were considered under surveillance if ≥ 2 HAdV blood tests were run in the first 14 days post-HCT. Published HAdV disease and attributable death definitions were applied to patients with positive HAdV PCR tests from any site and adjudicated by a central review committee. HAdV events were designated asymptomatic infection or possible, probable, or proven disease. HAdV DNAemia and disease incidence, time to event, and mortality and attributable case fatality rates were described.

**Figure:**
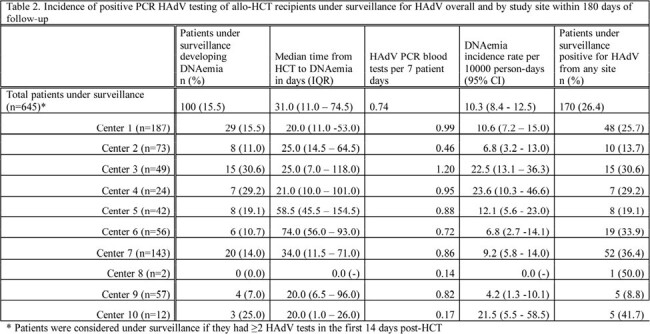

**Results:**

The cohort included 831 allo-HCT recipients: 645 under surveillance & 186 not (Table 1). 15.5% of patients under surveillance had DNAemia; median time to DNAemia was 31.0 days from HCT (Table 2). Probable/proven disease was documented in 11.9% and 3.8% of patients under and not under surveillance, with median time to disease 37.0 days and 66.0 days from HCT, respectively (Table 3). Overall mortality rates were 7.6% and 8.6% in patients under and not under surveillance (Table 4). Attributable case fatality rates for those with probable or proven disease were 13.0% and 14.3% in those under and not under surveillance, respectively.

**Figure:**
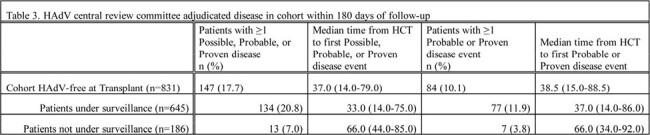

**Conclusion:**

HAdV infection and disease events were frequent; however, incidence was higher in patients under surveillance, suggesting detection bias. HAdV disease was identified as a frequent cause of death. Attributable case fatality rates were similar regardless of surveillance, suggesting early detection did not improve outcomes. Future analyses of these data will determine patient factors that identify allo-HCT patients at high risk for HAdV infection and disease to target for novel prophylaxis or pre-emptive therapy studies.

**Figure:**
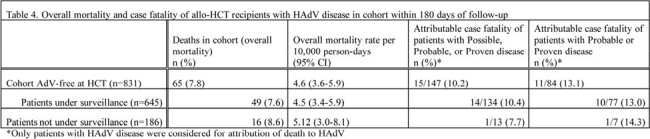

**Disclosures:**

**Michael D. Green, MD, MPH**, ADMA: Advisor/Consultant|Bristol Myers Squibb: Advisor/Consultant|ITB-MED: Advisor/Consultant|kamada: Honoraria **Monica I. Ardura, DO, MSCS**, Karius: Advisor/Consultant|Miravista: Grant/Research Support **Jeffrey Auletta, MD**, Ascella Health: Advisor/Consultant **Diego R. Hijano, MD, MSc**, FDA: Grant/Research Support|Merck: Grant/Research Support|National Institute of Health: Grant/Research Support **William J. Muller, MD**, Ansun Biopharma: Grant/Research Support|Astellas Pharma: Advisor/Consultant|Astellas Pharma: Grant/Research Support|AstraZeneca: Advisor/Consultant|AstraZeneca: Grant/Research Support|DiaSorin: Advisor/Consultant|DiaSorin: Honoraria|Eli Lilly and Company: Grant/Research Support|Enanta Pharmaceuticals: Advisor/Consultant|Enanta Pharmaceuticals: Grant/Research Support|F. Hoffmann-LaRoche Ltd (Roche): Grant/Research Support|Finley Law Firm, P.C.: Advisor/Consultant|Gilead Sciences: Grant/Research Support|Melinta Therapeutics, Inc.: Grant/Research Support|Merck Sharpe & Dohme: Grant/Research Support|Moderna: Grant/Research Support|Nabriva Therapeutics, plc: Grant/Research Support|Paratek Pharmaceuticals, Inc.: Grant/Research Support|Pfizer: Grant/Research Support|ProventionBio: Advisor/Consultant|Sanofi: Advisor/Consultant|Sanofi: Honoraria|Tetraphase Pharmaceuticals, Inc.: Grant/Research Support **Michael Grimley, MD**, SymBio Pharmaceuticals Limited: Advisor/Consultant **Lara A. Danziger-Isakov, MD, MPH**, Aicuris: clinical research contract, paid to institutio|Ansun BioPharma: clinical research contract, paid to institution|Astellas: Advisor/Consultant|Astellas: clinical research contract, paid to institutio|Merck: clinical research contract, paid to institutio|Pfizer: Grant/Research Support|Takeda: clinical research contract, paid to institutio **Brian T. Fisher, DO, MPH/MSCE**, Allovir: Grant/Research Support|Astellas: Data Safety Monitoring Board|Merck: Grant/Research Support|Pfizer: Grant/Research Support

